# *C. elegans ten-1 *is synthetic lethal with mutations in cytoskeleton regulators, and enhances many axon guidance defective mutants

**DOI:** 10.1186/1471-213X-10-55

**Published:** 2010-05-24

**Authors:** Catarina Mörck, Vivekanand Vivekanand, Gholamali Jafari, Marc Pilon

**Affiliations:** 1 Department of Cell and Molecular Biology, University of Gothenburg S-405 30 Gothenburg, Sweden; 2 Department of Molecular and Cell Biology, University of California, Berkeley, Berkeley, California 94720, USA

## Abstract

**Background:**

Teneurins are transmembrane proteins that assist morphogenetic processes in many organisms. *ten-1 *is the *C. elegans *teneurin homolog with two transcripts, *ten-1a *and *ten-1b*, that respectively encode a long (TEN-1L) and short (TEN-1S) form of the protein. We previously isolated a *C. elegans *mutant where one pharyngeal neuron was frequently misplaced, and now show that it corresponds to a novel allele of *ten-1*.

**Results:**

The novel *ten-1(et5) *allele is a hypomorph since its post-embryonic phenotype is weaker than the null alleles *ten-1(ok641) *and *ten-1(tm651)*. *ten-1 *mutants have defects in all pharyngeal neurons that we examined, and in vivo reporters show that only the long form of the *ten-1 *gene is expressed in the pharynx, specifically in six marginal cells and the M2 neurons. Defects in the pharyngeal M2 neurons were enhanced when the *ten-1(ok641) *mutation was combined with mutations in the following genes: *mig-14*, *unc-5, unc-51, unc-52 *and *unc-129*. None of the body neurons examined show any defects in the *ten-1(ok641) *mutant, but genetic interaction studies reveal that *ten-1(ok641) *is synthetic lethal with *sax-3, unc-34 *and *unc-73*, and examination of the hypodermal cells in embryos of the *ten-1(ok641) *mutant point to a role of *ten-1 *during hypodermal cell morphogenesis.

**Conclusions:**

Our results are consistent with *ten-1 *normally providing a function complementary to the cytoskeletal remodeling processes that occur in migrating cells or cells undergoing morphogenesis. It is possible that *ten-1 *influences the composition/distribution of extracellular matrix.

## Background

Teneurins are transmembrane proteins that participate in morphogenetic processes in many organisms [1,2]. Teneurins have a single transmembrane domain, a very large and cleavable extracellular domain containing eight EGF repeats, four NHL domains and more than 20 YD repeats, as well as a cleavable intracellular domain (ICD) that can be translocated to the nucleus. The *Drosophila *homologs, *Ten-m *and *Ten-a*, are the only pair-rule genes that do not encode traditional transcription factors [3-5]. Instead, they act at the cellular blastoderm stage, and cleavage of the ICD may allow it to directly regulate the transcription of target genes in alternate parasegments. *Drosophila Ten-m *is also important for several other developmental processes, including retina development [6], and peripheral nervous system development in imaginal disc-derived organs [7]. In vertebrates, the *teneurin *genes are expressed most prominently in developing neuronal tissues and are important for neuronal patterning and axon guidance [1,2,8]. The distinct expression profiles of various teneurins or teneurin isoforms in vertebrates, together with the neuronal defects observed in mutants, strongly suggest that teneurins act during cell communication to influence neurite outgrowth and guide axons [1,8,9]. Biochemical studies on the four mouse teneurins (Ten-m1 to Ten-m4) have shown that the EGF domains are important for teneurin homo- or heterodimerization via covalent disulfide links between the second and fifth EGF repeats [10,11], while the NHL and YD repeats form large glycosylated globular domains that may mediate homotypic or heterotypic interactions between cells that express the same or different forms of teneurin, as well as interactions with the extracellular matrix [1,2].

**Figure 1:**
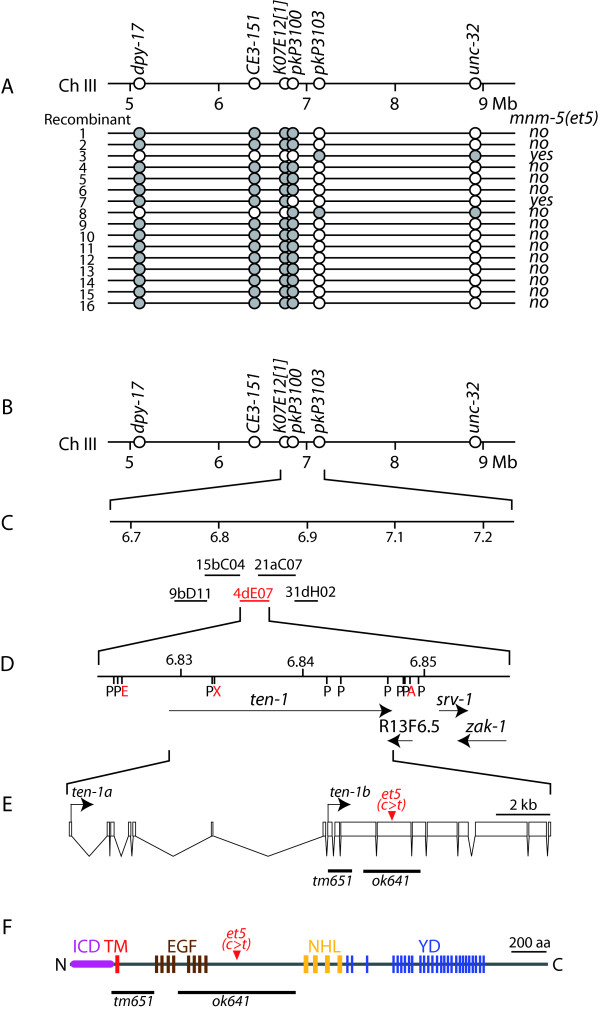
**Mapping of *ten-1(et5)***. (A) Shows part of the map for chromosome III, including the positions of the genetic markers used to map *mnm-5/ten-1(et5)*. Also shown are the structures of 16 informative recombinants among the F2 progeny of *dpy-17 ten-1(et5) unc-32 *mutants crossed with the Hawaiian strain CB4860. Open circles refer to the N2 allele at each locus. (B) and (C) Show an enlarged region of chromosome III and the locations of five fosmids that were tested for their ability to rescue the *ten-1(et5) *mutant. The full name for each fosmid includes the prefix "WRM06", and the rescuing fosmid WRM064dE07 is highlighted in red. (D) Partial restriction map of the WRM064dE07 fosmid, which contains the genes *ten-1*, *srv-1*, *zak-1 *and *R13F6.5*. The restriction enzyme sites AhdI, EcoNI, PstI and XhoI sites are respectively indicated as A, E, P and XhoI. (E) Structure of the *ten-1 *gene showing the two transcription starts, the regions deleted in the *tm651 *and *ok641 *alleles and the position of the *et5 *c>t nucleotide substitution. (F) Domain structure of the TEN-1 protein.

In *C. elegans*, the *ten-1 *gene can be transcribed from two distinct promoters to produce the transcripts *ten-1a *and *ten-1b*, which respectively encode two isoforms of the protein: TEN-1L, which contains all the teneurin domains described above (see Fig. 1F), and TEN-1S, which lacks the ICD but contains the rest of the protein including the transmembrane domain. Two null alleles of *C. elegans ten-1 *have previously been isolated using a reverse genetics approach. These *ten-1 *mutants are reported to have defects in the growth of several axons and in the development of the gonads and epidermis, and these defects correlated with disruptions in the extracellular matrix (ECM). These observations, together with the fact that *ten-1 *acts redundantly with genes encoding ECM components or adhesion molecules, such as integrin alpha (*ina-1*), dystroglycan (*dgn-1*), laminin alpha beta (*epi-1*) and nidogen (*nid-1*), have led to the conclusion that teneurin in *C. elegans *may have its primary function as an ECM organizer [2,12,13].

In the present study we provide novel insights into the functions of *ten-1*. Specifically we provide a detailed characterization of its embryonic expression, identify the expressing cells within the pharynx, show that *ten-1 *contributes to the development of several pharyngeal axons, and identify several genes with which *ten-1 *interacts. We also take advantage of the novel nature of the *ten-1(et5) *mutant, i.e. truncation just after the EGF domains, to draw structure-function conclusions.

## Results

### *mnm-5(et5) *is a novel allele of *ten-1*, and is therefore renamed *ten-1(et5)*

We previously isolated the *mnm-5(et5) *mutant in a forward genetics screen for mutants with abnormal M2 neurons [14], and later also found that it causes defects in the pharyngeal neurons NSMR and NSML [15]. By genetic mapping using recombinants between visible genetic markers, then between single nucleotide polymorphisms that distinguish the parental N2 strain from the Hawaiian strain CB4856, we defined the position of the *mnm-5 *gene to within a narrow region of chromosome III (Fig. 1A). We then tested five fosmids covering the genetic area of interest for their ability to rescue the *mnm-5(et5) *mutant (Fig. 1B-C). One fosmid, WRM064dE07, scored positive in this assay. From that fosmid we cut and purified an AhdI-EcoNI restriction fragment that contains the *ten-1 *gene but no other complete gene (Fig. 1D). This fragment also rescued the *mnm-5(et5) *mutant, showing that the mutation corresponds to a novel allele of the *ten-1 *gene. This was confirmed by sequencing: the *et5 *allele corresponds to a c>t point mutation that introduces a stop codon just downstream of the 8 EGF repeats in the extracellular domain of the TEN-1 protein (Fig. 1E-F). *mnm-5(et5)*, which will henceforth be referred to as *ten-1(et5)*.

### *ten-1(et5) *is not a null allele

Two deletion mutant alleles of *ten-1*, i.e. alleles *ok641 *and *tm651*, have been characterized previously (see Fig. 1F). The *ok641 *allele is in frame and supports expression of a transcript [12] and the *tm651 *allele has an internal deletion of 890 base pairs that introduces a frameshift early in the coding sequence [13]. Both the *ok641 *and *tm651 *mutations are considered to be functional null alleles [13]. In their homozygous states these mutant alleles cause severe phenotypes including embryonic (~6%) and larval (~30%) lethality, and sterile adults or adults with vulva defects (17%), with less than 45% of L1s growing into fertile adults (Table 1). By comparison, we found that the novel *ten-1(et5) *allele exhibits the same rate of embryonic lethality as the null alleles (~6%), but reduced incidence of post-embryonic phenotypes, such that over 70% of homozygous *et5 *progeny grow into fertile adults (Table 1). *ten-1(et5) *is therefore not a null allele, which suggests an important function for the four EGF repeats present in the *ten-1(et5) *allele but absent from the *tm651 *and *ok641 *alleles (see Fig. 1F).

**Table 1:** Characterization of visible phenotypes in *ten-1 *mutants.

Genotype	Emb* (%)	Lvl* (%)	Vul/Ste* (%)	Fer* (%)	n
N2	0	0	0	100	207
*mnm-5(et5)*	5.8	18.6	2.5	73.1	191
*ten-1(ok641)*	6.1	30.9	18.3	44.7	211
*ten-1(tm651)*	6.5	33.8	16.1	43.5	186

*Emb: embryonic lethal; Lvl: larval lethal; Vul/Ste: vulva defective or sterile adult; Fer: fertile adult.

### Expression of *ten-1 *transcriptional reporters

Others have reported on the expression profile of the two *ten-1 *isoforms in *C. elegans *[12,13]. We independently generated *ten-1a::gfp *and *ten-1b::gfp *transcriptional reporters and analyzed their expression during development and in adults. Our observations are generally consistent with the published ones. However, we also paid careful attention to embryonic and pharyngeal expression and made the following novel observations.

**Figure 2:**
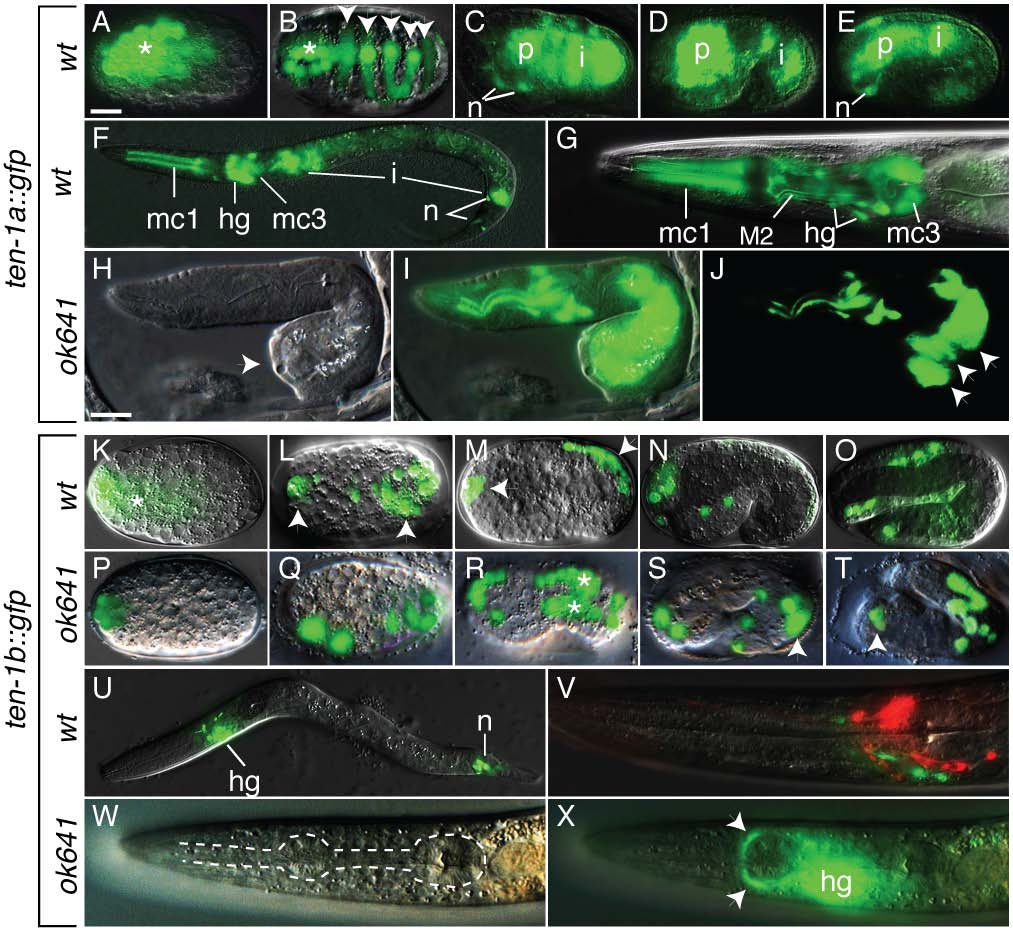
**Expression of *ten-1a/b *transcriptional reporters**. (A-E) Embryonic expression of *ten-1a::gfp*. The asterisk in (A) indicates the anterior cluster of GFP-positive cells in a ~150 min embryo. Arrowheads in (B) indicate intercalating hypodermal cells, while the asterisk indicates a cluster of pharyngeal precursor cells in a ~300 min embryo. (C-E) Shows that pharyngeal cells (p), intestinal (i) and some neurons (n) express *ten-1a::gfp *in ~350 min (dorsal view), 1.5-fold, and 2-fold stage embryos, respectively. (F-G) Show a L1 larva and the head of an adult, respectively. Note expression in the marginal cells (mc1 and mc3), head ganglion neurons (hg), the intestine (i) and the M2 pharyngeal neuron (M2). (H-J) Show a L1 *ten-1(ok641) *mutant larva expressing *ten-1a::gfp*. Note the deformed posterior end (indicated by arrowhead in H), and the poor connection between the posterior intestinal cells (indicated by arrowheads in J). (I) is an overlay of (H) and (J). (K-O) Embryonic expression of *ten-1b::gfp*. The asterisk in (K) indicates the anterior cluster of GFP-positive cells in a ~150 min embryo. Arrowheads in (L-M) indicate anterior and posterior clusters of hypodermal cells in ~300 min embryos shown from a dorsal or lateral perspective, respectively. (N-O) *ten-1b::gfp *expression in several neurons of the head and tail in 1.5 and 2-fold embryos, respectively. (P-T) Show expression of *ten-1b::gfp ten-1(ok641) *mutant embryos. Note abnormal development of the embryos and the persistence of strong GFP expression in dorsal hypodermal cells (asterisks in R; arrowheads in S and T) at stages where the expression is declining or lost from wild-type embryos. (U) Shows a L1 larva, with several head ganglion neurons (hg) and tail neurons (n) expressing GFP. (V) Shows an adult pharynx expressing *ten-1b::gfp *and stained with DiI, which labels the taste sensory amphid neurons (red). Note the absence of overlap between the DiI and GFP signals. (W-X) Show the head of an adult *ten-1(ok641) *mutant expressing *ten-1a::gfp*. Note the presence of GFP-positive axons forming an ectopic nerve ring anterior to the metacorpus (arrowheads), which is never seen in wild-type controls. The scale bar in (A) represents 10 μm and applies to all panels except (H-J), which have a separate scale bar.

In wild-type embryos, *ten-1a::gfp *is first expressed in a cluster of cells in the anterior half at approximately 150 minutes after fertilization (Fig. 2A). These cells are precursors to the hypodermal cells, which are evident at 300 minutes post-fertilization, when the cells intercalate and begin the process of ventral closure (Fig. 2B), and to pharyngeal and intestinal cells, which are evident beginning at the bean stage (Fig. 2C). In later stages, strong expression of *ten-1a::gfp *persists in pharyngeal and intestinal cells, and appears in several head neurons (Fig. 2D-E). Examination of L1 larvae and adults allowed us to identify 8 pharyngeal cells that express *ten-1a::gfp*: the three marginal cells mc1, the three marginal cells mc3, and the neurons M2L and M2R (Fig. 2F-G). As reported previously, adults also express *ten-1a::gfp *in vulva muscles, the gonad distal tip cells, the intestine, several tail neurons including DVB and some other cells ([12]; data not shown).

The expression of the *ten-1b *reporter differs from that of *ten-1a*. Initial expression is detected in fewer anterior cells at 150 minutes post-fertilization (Fig. 2K), and becomes restricted to anterior neuronal cells and posterior hypodermal cells by 300 minutes (Fig. 2L-M). By the 1.5-fold stage (~460 minutes post-fertilization), hypodermal cell expression gradually fades away, and strong expression is found only in neurons of the head (Fig. 2N). This pattern persists to the end of embryogenesis (Fig. 2O). As previously reported, post-embryonic expression is found in head and tail neurons and some other cells (Fig. 2U; [12]). We used DiI to show that the *ten-1b::gfp *positive neurons are not amphid or phasmid neurons since they did not pick up the dye (Fig. 2V). Expression of the *ten-1b *reporter is never observed within the pharynx.

### *ten-1 *reporters help visualize mutant phenotypes

The expression of *ten-1a::gfp *and *ten-1b::gfp *was examined in the *ten-1(ok641) *null mutant background to try and detect expression changes or morphogenesis defects in the *ten-1 *expressing cells. Mutant embryos usually did not differ from wild-type until the bean stage. After this stage, ~6% of the *ten-1(ok641) *mutant embryos failed to complete hypodermal cell intercalation and also failed to fold properly their posterior end, such that the 1.5- and 2-fold stages were delayed and poorly formed. The observed defects in hypodermal cell intercalation in mutant embryos coincides with persistent strong expression of *ten-1b::gfp *in dorsal hypodermal cells well beyond the developmental stages at which it becomes barely detectable in control embryos (Fig. 2P-T).

Mutant animals that survived through embryogenesis exhibit phenotypes easily visualized using the *ten-1 *transcriptional reporters. Firstly, many L1 larvae have deformations in their posterior half, and the *ten-1a::gfp *reporter reveals that the intestinal cells in these larvae are misshapen and poorly bound to one another; this phenotype may account for the 30% embryonic lethality observed in this mutant (Fig. 2H-J). Secondly, and as previously reported [12], many of the escapers that grow into healthy-looking adults actually have gross defects in the anatomy of their vulva muscles and often fail to open a vulva slit (data not shown). These defects in vulva morphogenesis likely contribute to the sterility/exploded vulva phenotypes observed at a frequency of 15-20% in the *ten-1(ok641) *mutant background. Expression of the *ten-1b::gfp *reporter was generally normal in the *ten-1(ok641) *mutant escapers that matured into L1s without posterior defects. However, we noted a low incidence of animals exhibiting misplaced axon trajectories within the head (~2%; N = 400), such that they appeared as a separate nerve ring just anterior to the pharyngeal metacorpus (Fig. 2W-X).

In summary, cells that express the *ten-1 *gene embryonically or post-embryonically often exhibit developmental defects in the *ten-1(ok641) *mutant background. This result suggests that *ten-1 *may generally act cell-autonomously.

### Pharyngeal neuron defects in *ten-1 *mutants

**Figure 3:**
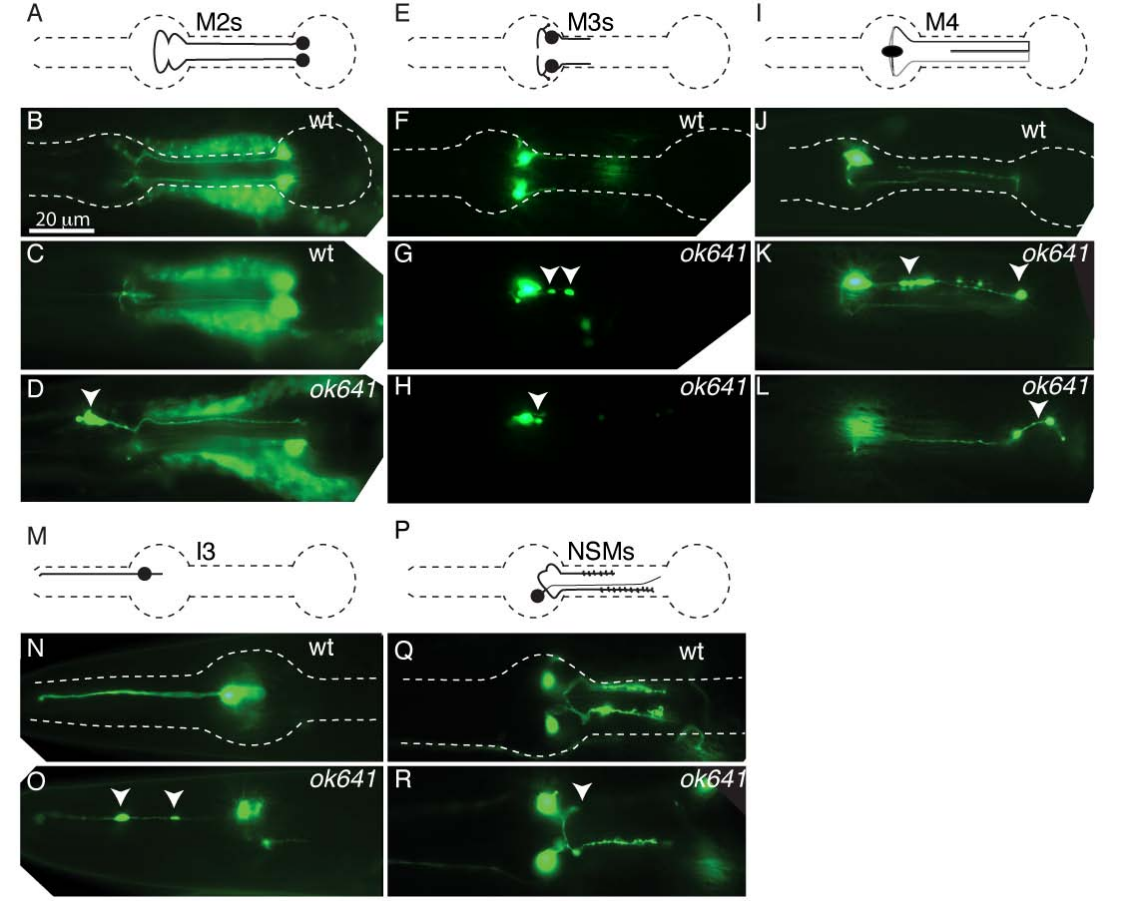
**Pharyngeal neuron defects in *ten-1 *mutants**. Each series shows the following: a cartoon representation of the normal morphology of the examined neuron, photographs of the wild-type neurons visualized with a specific GFP reporter (N2; see materials and methods), and mutant animals (*ok641*) with typical defects indicated by arrowheads. (C) Shows a different focal plane of the worm shown in (B) to make the ends of the distal M2s visible. The arrowheads indicate a misplaced M2 neuron cell body (D), conspicuous varicosities (G, K, O), ectopic branches (H), misguided axons (L) or truncated axons (R). The scale bar in (B) represents 20 μm and applies to all photographs.

As mentioned earlier, the *ten-1(et5) *mutant was isolated by virtue of its M2 pharyngeal neurons defects [14], and later also found to have defects in the neurons NSMR and NSML [15]. To determine if other pharyngeal neurons depend on functional *ten-1 *for their development, we examined the trajectories of the pharyngeal neurons for which we could obtain specific GFP reporters in both the *ten-1(et5) *and the more severe *ten-1(ok641) *mutants. Specifically, we examined the M1, M2, M3, M4, I3 and NSM neurons, and found that *ten-1 *contributes to the guidance of all these neurons, but that its role varies in importance (Fig. 3 and Table 2). The most typical defects are the presence of large varicosities and truncations of the axons, which are both indicative of growth cones stalling during axon elongation. Misguided axon trajectories are also observed with variable frequencies in the motor neurons M2, M3 and M4. Of the neurons examined, M4 has the most complex trajectory and showed the strongest requirement for functional *ten-1*, with more than 60% of neurons being defective in the *ten-1(et5) *and *ten-1(ok641) *mutant backgrounds. Only the M2 neuron exhibited misplaced cell bodies, with a frequency of roughly 10%, as previously reported. The two alleles examined, *et5 *and *ok641*, produced a very similar range of phenotypes with similar severities and penetrance.

**Table 2:** Scoring of pharyngeal neurons in the *ten-1(et5) *and *ten-1(ok641) *mutants.

Neuron	Genotype	Normal %	Varicosities %	Truncated %	Misguided %	Others *	n
M2	wt	99.8			0.2		419
	*et5*	87.7		0.2	3.9	8.3	432
	*ok641*	86.9		1.3	3.3	8.5	459
							
M3	wt	100					130
	*et5*	60.3	5.7		13.6	20.4	191
	*ok641*	60.8			15.2	24.0	216
							
M4	wt	100					114
	*et5*	58	30.4		5.8	5.8	102
	*ok641*	34.9	42.5		11.0	11.6	181
							
I3	wt	99.1	0.9				222
	*et5*	89.6	10.3				203
	*ok641*	92.0	8.0				210
							
NSM	wt	98.9		1.1			271
	*et5*	82.5	6.6	10.9			241
	*ok641*	79.6	7.8	12.6			165

Notes

* M2 neuron: indicates the percentage of M2 neurons that were misplaced into the isthmus or metacorpus.

* M3 neuron: indicates cases of missing M3 neuron cell bodies.

* M4 neuron: indicates supernumerary ectopic outgrowths.

### *ten-1(ok641) *mutants have no obvious body neuron defects

Chiquet-Ehrismann and co-workers previously reported neuronal defects outside the pharynx in animals treated with RNAi against *ten-1 *as well as in *ten-1(ok641) *mutant animals [12]. This surprised us in view of the fact that our initial description of the *ten-1(et5) *mutant had not revealed any such defects [14] and because the viable *ten-1 *mutant individuals are not uncoordinated. We used several neuronal reporters to examine carefully whether *ten-1 *is important for the development of neurons outside of the pharynx. First, we introduced a pan-neuronal GFP reporter, i.e. *evIs111*, in the *ten-1(ok641) *mutant. We detected no obvious abnormalities in the extrapharyngeal nervous system, including the positioning of lateral body neurons, and the trajectories of several lateral neurons that can be seen using *evIs111 *(Additional file 1: fig. S1 A-B). Similar results were obtained with the other presumed null mutant, *ten-1(tm651) *(data not shown). We also examined carefully the body neurons that express *ten-1b::gfp *and found that these too were normal in the *ten-1(ok641*) background, except for the ~2% of larvae that had mispositioned nerve ring axons to positions anterior of the metacorpus (Fig. 2X). The six microtubule-rich mechanosensory neurons were also specifically scored in *ten-1(ok641) *mutants, this time using a *mec-7::gfp *reporter. Four of these neurons (ALML, ALMR, PLML and PLMR) project on the lateral side of the body, while the two others (AVM and PVM) originate from the lateral side, navigate ventrally into the nerve cord then project anteriorly [16,17]. No defects were observed in the trajectories of these six neurons (Additional file 1: fig. S1 C-D). Finally, we obtained control and *ten-1(ok641) *strains carrying a *ten-1b::gfp *reporter (a kind gift from R. Chiquet-Ehrismann; [12]) and scored these for possible neuronal defects in the GFP-positive neurons. Again we observed no neuronal defects in randomly picked L4 or adult worms (Additional File 1: Fig S2 A-D), except in those rare instances (<3%; N > 100) where a viable worm also exhibited obvious morphological defects (Additional file 1: fig. S2E). From our analysis of various neurons in *ten-1 *mutants, we conclude that *ten-1 *is not generally important for neuronal development (e.g. no gross defects in extrapharyngeal neuroanatomy or in specific mechanosensory neurons), but rather is specifically required by some neurons (e.g. several pharyngeal neurons). It seems likely that any neuronal defects in body neurons are secondary to body morphogenesis defects.

### Enhanced body muscle expression of *unc-129 *in *ten-1(ok641) *mutants

**Figure 4:**
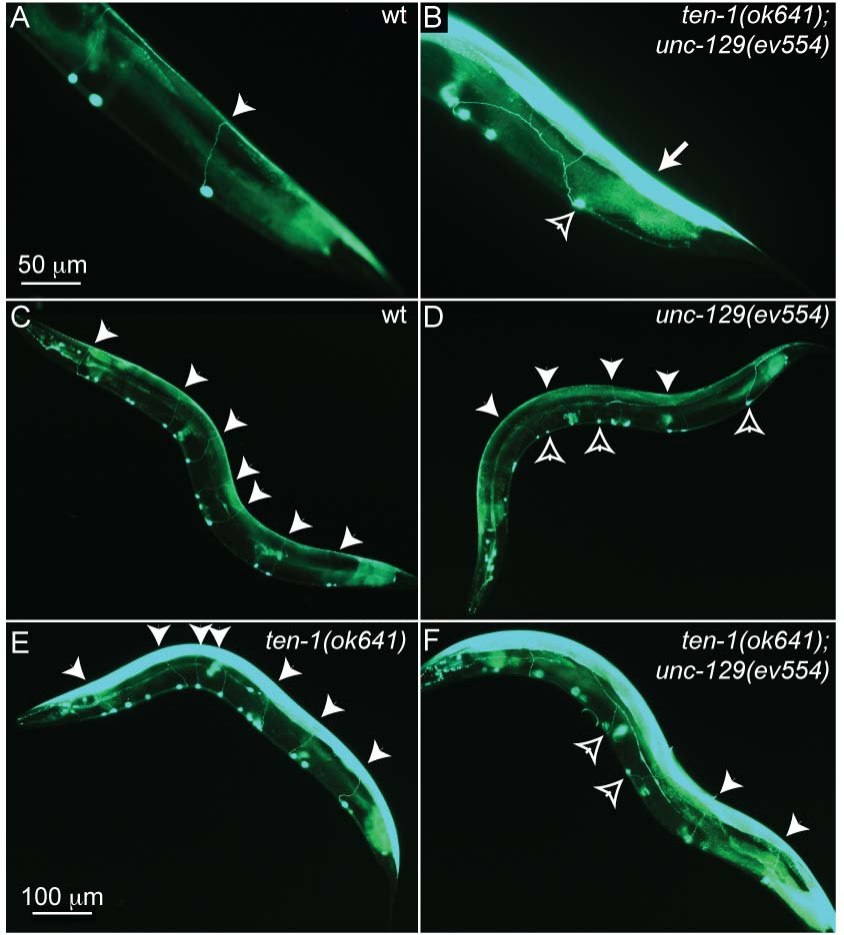
***unc-129::gfp *expression in the *ten-1(ok641) *and *unc-129(ev554) *mutants**. All panels show worms transgenic for a *unc-129::gfp *reporter expressed in the DA/DB neurons and in the dorsal muscle cells. (A) Shows a control worm in which one clear circumferential axon is in indicated by the filled arrowhead. (B) Shows a *ten-1(ok641);unc-129(ev554) *double mutant where the same axon errs and branches abnormally (open arrowhead), and the dorsal muscle expression is dramatically enhanced compared to control (arrow). (C-D) Show lower magnification views of animals with the indicated genotypes. Filled arrowheads indicate axons that projected correctly circumferentially, while open arrowheads indicate axons that erred and branched abnormally, failing to reach the dorsal side. The scale bar in (A) applies also to (B), while the scale bar in (C) applies to panels (C-F).

To further test the possibility that some *C. elegans *body neurons may rely on *ten-1 *during their development, we introduced an *unc-129::gfp *reporter in *ten-1(ok641) *mutant animals. This reporter in expressed in the DA/DB neurons that project circumferentially from the ventral cord to innervate the dorsal body muscles, and also weakly in the dorsal muscle cells [18]. Consistent with the lack of any obvious Unc phenotype, no defects were observed in the DA/DB neurons of *ten-1(ok641) *mutants. Unexpectedly however, we discovered a 20-fold increase in the expression levels of the reporter in the dorsal muscle cells of mutants, as judged by the intensity of the GFP signal (Fig. 4). It seemed possible that the muscle cells compensate for *ten-1 *loss by expressing and secreting more of the *unc-129 *guidance signal. This possibility prompted us to investigate the trajectories of the DA/DB neurons in a *ten-1(ok641);unc-129(ev55) *double mutant background. As shown in Fig. 4F, the double mutant shows the same frequency of DA/DB defects as in the *unc-129 *single mutant, suggesting that *ten-1 *does not contribute at all to the guidance of these axons, although it obviously plays a role in regulating their level of TGF-β expression from the *unc-129 *locus.

### *ten-1 *enhances the severity of mutations affecting growth cone and/or cytoskeleton regulators

The pharyngeal neuron defects in the *ten-1 *mutant are never 100% penetrant even in the most severely affected neurons, which indicate redundancy with other genes and pathways. In our previous study of the M2 neurons we discovered that mutations in the *sax-3, unc-5 and unc-6 *genes cause misplacement of the M2 neurons similar to those seen in *ten-1(et5) *but at a much lower penetrance [14]. To explore if *ten-1 *acts in or in parallel with netrin, ROBO or other pathways, we performed a genetic interaction study. The mutations tested are listed in Table 3. Single and double mutants were scored for viability and for M2 neuron trajectories, and the results are presented in Table 4.

**Table 3:** Lists of genes tested for possible interaction with *ten-1*.

*Gene(allele)*	Allele type	Function/pathway
*mig-14(ga62)*	hypomorph	Wnt-secretion factor
*sax-3(ky123)*	null	receptor/robo slit
*slt-1(e15)*	null	ligand/robo slit
*unc-5(e53)*	null	receptor/netrin
*unc-6(ev400)*	null	ligand/netrin
*unc-34(e315)*	nonsense	Enabled VASP homolog/netrin and robo
*unc-40(e271)*	null	receptor/netrin
*unc-51(e369)*	dominant negative	serine threonine kinase
*unc-52(e1421)*	possible null	perlecan homolog
*unc-73(e396)*	null	guanine nucleotide exchange factor
*unc-129(ev554)*	null	secreted TGF-β

**Table 4:** Pharyngeal M2 neuron defects in *ten-1 *mutants.

Genotype	wt (%)	Misplac (%)	Ipsil (%)	Trunc (%)	Other (%)	N
*etIs2*	99.8		0.2			419
*mnm-5(et5); etIs2*	87.7	8.3	3.9	0.2		432
*ten-1(ok641); etIs2*	86.9	8.5	3.3	1.3		459
*ten-1(tm651); etIs1*	83.3	14.6	2.0			246
*mig-14;etIs2*	85.5	0.9	0.4	3.0	10.3	234
*mig-14;etIs2 ok641*	36.4	26.6	8.9	12.1	15.9	214
*sax-3; etIs2*	68.9	5.1	7.2	2.9	15.7	235
*sax-3; etIs2 ok641*	LETHAL					-
*slt-1; etIs2*	97.3		1.9	0.8		262
*slt-1; etIs2 ok641*	93.9	1.1	3.0	1.9		264
*unc-5; etIs2*	76.8		16.0	7.2		250
*unc-5; etIs2 ok641*	35.6	6.2	42.9	15.3		177
*unc-6; etIs2*	23.7	0.4	37.9	37.9		253
*unc-6; etIs2 ok641*	42.2	6.6	42.2	8.3	0.6	469
*unc-34; etIs2*	91.8		4.9	3.3		245
*unc-34; etIs2 ok641*	LETHAL					-
*unc-40; etIs2*	78.3		6.5	14.8	0.4	230
*unc-40; etIs2 ok641*	78.6	2.5	10.7	8.2		280
*unc-51; etIs2*	83.7		5.9	10.4		270
*unc-51; etIs2 ok641*	49.2	7.4	33.5	9.7		268
*unc-52; etIs2*	94.4		3.7	1.8		215
*unc-52; etIs2 ok641*	40.8	7.1	42.9	9.2		184
*unc-73; etIs2*	50.4		19.9	29.7		236
*unc-73; etIs2 ok641*	LETHAL					-
*unc-129; etIs2*	91.7		2.0	6.3		204
*unc-129; etIs2 ok641*	74.1	8.6	8.2	9.1		220

Notes

wt: normal

Misplac: M2 cell body mispositioned into the metacorpus

Ipsil: ipsilateral outgrowth

Trunc: truncated distal end

Contra: contralateral outgrowth

Post: posterior outgrowth

Other: Contralateral outgrowths in the case of *sax-3;etIs2, unc-6; etIs2 ok641 *and *unc-40; etIs2*. Ectopic dorsal branch, posterior branch, misplaced cell into isthmus, growth posterior from metacorpus back into isthmus in the case of *mig-14; etIs2 *and *mig-14;ok641 etIs2*.

**Figure 5:**
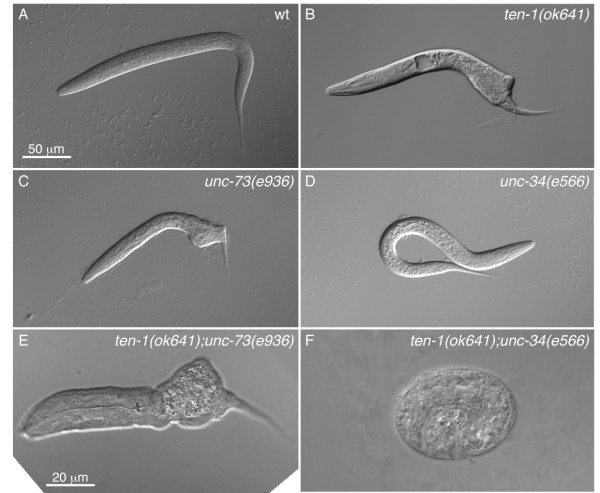
**Emb and Lvl synthetic phenotypes involving *ten-1 *mutants**. Larvaes of the indicated genotypes are shown. Note that the posterior defect seen in the *ten-1(ok641) *and *unc-73(e936) *single mutants have low penetrance (<5%) while the defects in the two shown double mutants (E-F) are 100% penetrant. Scale bar in (A) applies to all panels.

A striking observation concerns the synthetic embryonic or L1 larval lethality obtained when combining the *ten-1(ok641) *mutation with mutations in the *sax-3(ky123)*, *unc-34(e315) *or *unc-73(e396) *genes (see Fig. 5). The synthetic lethality demonstrates important roles for these genes in early embryonic processes, and likely reflects the complementarity between the ECM environment to which *ten-1 *contributes [13], and the cytoskeletal changes regulated by *sax-3*, *unc-34 *and *unc-73 *in response to that environment as embryonic cells migrate, adhere and otherwise interact with each other.

Another striking finding is that the hypomorphic mutation *mig-14(ga62) *and the null mutation *ten-1(ok641) *synergistically enhanced their effects on M2 cell position and axon trajectories (Table 4). In particular, the double mutant had over 25% misplaced M2 cell bodies, the highest incidence ever observed for any mutant or double mutant. *mig-14 *encodes the sole *C. elegans *homolog of the Wnt-secretion factor Wntless, and it plays an important role in anterior-posterior guidance during cell migration and axon elongation [19,20]. Our results suggest that *ten-1 *is also important for these processes, acting either in parallel with *mig-14 *or by increasing the activity of the *mig-14 *pathway.

With regards to the distal M2 ends, which are dependent on growth cones for their development [14], we found that most mutations tested increased the occurrence of defects when combined with the *ten-1(ok641) *mutation, especially in the ipsilateral outgrowth class that is characterized by the distal ends failing to migrate dorsally within the metacorpus, erring instead anteriorly in their original focal plane (Table 4). The most dramatic genetic interactions that affected ispilateral outgrowth involved *ten-1(ok641) *together with *unc-51 *(a serine/threonine kinase important for the localization of guidance receptors; [21]) or with *unc-52 *(which encodes the *C. elegans *perlecan; [22,23]): while the single mutants individually displayed about 5% ipsilateral outgrowth, over 30% of the M2 neurons showed this defect in the double mutants (Table 4).

### *ajm-1::GFP *reveals a morphogenesis defect during embryogenesis in *ten-1 *mutants

**Figure 6:**
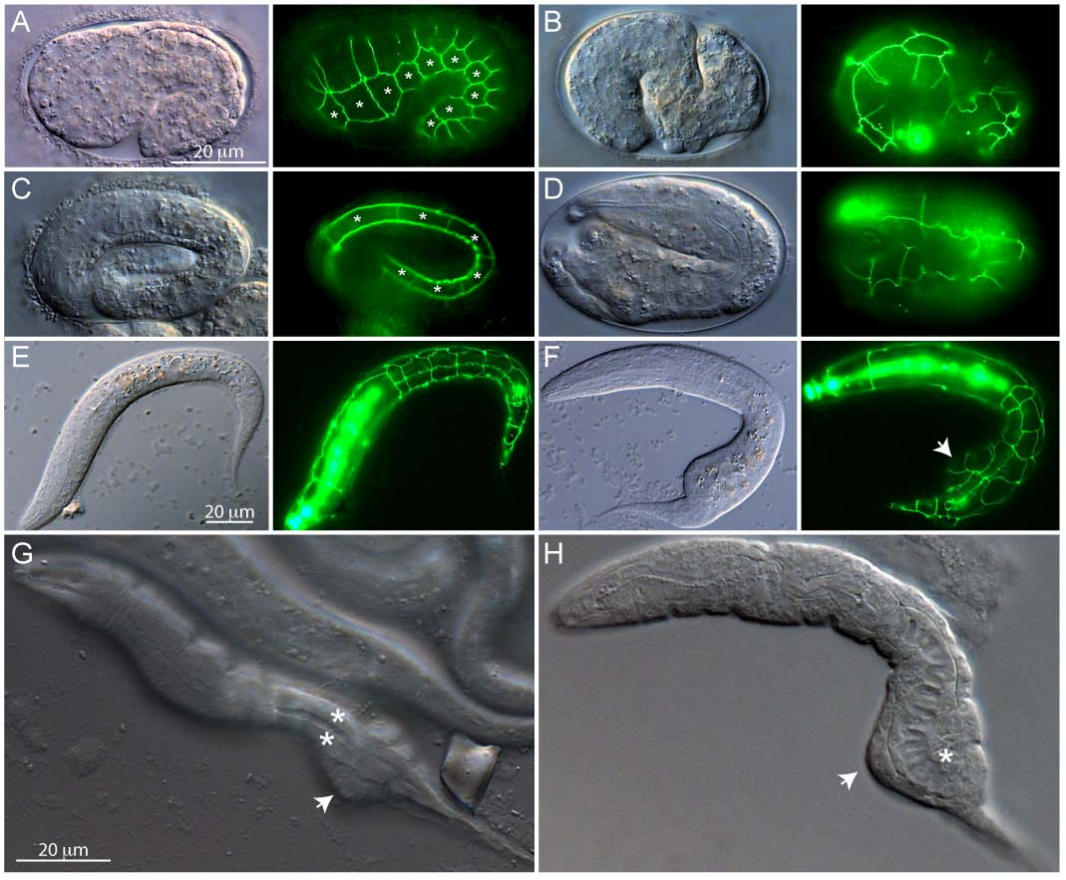
**Hypodermal cell defects in *ten-1(ok641) *embryos**. All animals shown are *ten-1(ok641) *mutants carrying the *ajm-1::gfp *transgene, which allows the visualization of the adherence junctions that surround the hypodermal cells. (A) A 1.5-fold embryo that is developing normally. Note the seam cells indicated by asterisks, and the ongoing hypodermal cell fusions on the dorsal side (discontinuous lines of GFP expression). (B) An example of a 2-fold embryo in which the posterior half has developed abnormally. Note the poorly organized pattern of *ajm-1::gfp *expression. (C) An early 3-fold stage embryo that is developing normally. Note the evenly spaced seam cells (asterisks) and the symmetrical distribution of the *ajm-1::gfp *pattern. (D) An early 3-fold embryo in which the posterior half has developed abnormally. Note in particular the meandering and disorganized pattern of *ajm-1::gfp *distribution. (E) Ventral aspect of an L1 larva that has developed normally. Again, note the regular shapes of the hypodermal cells. (F) Ventral aspect of an L1 larva with a deformity in its posterior half, which corresponds to a misshaped hypodermal cell (arrow). (G) DIC image of a *ten-1(ok641) *L1 larva with focus on the two muscle quadrants with normal appearance (asterisks) in spite of the posterior deformity (arrow). (H) DIC image of a *ten-1(ok641) *L1 larva with focus on the deformed intestine (asterisks) and posterior deformity (arrow).

The embryonic/larval lethality observed in several double mutants involving *ten-1(ok641) *strongly suggests an important role for *ten-1 *during early development (see Table 4 and Fig. 5). The expression of *ten-1 *reporters in the hypodermal cells of early embryos (Fig. 2 and 3) prompted us to examine the behavior of these cells in the *ten-1(ok641) *mutant using a marker of adherance junctions, i.e. *ajm-1::GFP*, to visualize the hypodermal cells as they intercalate and change shape during ventral closure and embryonic elongation. The parental strain SU93, which carries the *ajm-1::GFP *transgene, occasionally exhibits early embryonic arrest, mostly at pre-bean stages, at a frequency of 1.8% (N = 281 bean to 2-fold stage embryos scored), indicating that the transgene in itself may have some effect on embryogenesis. When the same transgene is introduced in the *ten-1(ok641) *background, embryonic phenotypes are evident at a frequency of 6.3% (N = 268 bean to 2-fold stage embryos scored), suggesting that *ten-1(ok641) *is responsible for about 4.5% embryonic defects, which is consistent with the study of the single mutant described earlier (Table 1). *ten-1(ok641) *embryos also exhibited phenotypes not seen in the parental strain SU93. In particular, posterior morphogenesis defects at the comma or later stages were specific for the *ten-1(ok641) *mutants (Fig. 6). The hypodermal cells are grossly disorganized in some embryos (Fig. 6B and 6D), while in others they failed to fuse or became mispositioned such as to cause bulges in the posterior half (Fig. 6F). These results are in general agreement with published work [12]. When larvae with deformed posterior halves are carefully examined by DIC microscopy, it was obvious that their muscle quadrants have developed successfully (Fig. 6G), but that their intestinal cells are grossly malformed (Fig. 6H).

## Discussion

### The *ten-1(et5) *allele is a hypomorph

Our observations complement those of Chiquet-Ehrismann and co-workers [12,13]. In their studies, they described the expression profiles of the *ten-1a *and *ten-1b *forms of the gene, the phenotypes induced by RNAi against *ten-1*, and the phenotypes of two null mutant alleles (*ok641 *and *tm651*). They also documented defects in basement membranes in the mutants, such as abnormal or deficient laminin distribution around the pharynx and developing gonad, and demonstrated that *ten-1 *acts in part redundantly with genes encoding the extracellular matrix components dystroglycan (*dgn-1*) and laminin (*epi-1*), and one integrin adhesion molecule (*ina-1*).

Our study adds several novel observations that will each be discussed separately. Firstly, we isolated a novel allele of *ten-1*, namely the *et5 *allele. The novel *et5 *allele is hypomorphic since it causes a milder post-embryonic phenotype than either the *ok641 *or *tm651 *alleles, which are both considered functional null alleles [13]. The TEN-1 protein expressed from the *ten-1 *allele will be truncated after the eight extracellular EGF domains. The null *ok641 *allele is predicted to encode a protein truncated between the fourth and fifth EGF domain. Our observations suggest that important post-embryonic functions may reside within the four EGF domains present in the *et5 *allele but absent from the *ok641 *allele. The second and fifth EGF repeats are important for homo- and hetero-dimerization [10,11], and the *et5 *allele is therefore expected to be able to multimerize. Because the *et5 *allele exhibits embryonic phenotypes with the same penetrance and severity as the null alleles, it seems possible that some embryonic functions of the TEN-1 protein are dependent on motifs located on the C-terminal side of the EGF domains. The NHL and YD repeats present in that part of the protein are expected to mediate homotypic or heterotypic interactions between cells that express teneurin, as well as interactions with the extracellular matrix [1,2]. These functions are critical during morphogenetic events and may also account for the roles of *ten-1 *in regulating extracellular deposition and composition, to which *ten-1 *clearly contributes [13].

### *ten-1 *may act cell-autonomously during the morphogenesis of hypodermal and vulva muscle cells

Both *ten-1a *and *ten-1b *are expressed in hypodermal cells during early embryonic development, and that expression ends by the time that these cells have completed their elongation, intercalation and ventral closure. An interesting observation is that the expression of *ten-1b *is enriched in the posterior hypodermal cells, the same cells that will show the most common morphogenesis defects in the *ten-1 *mutants. Similarly, *ten-1a *is expressed post-embryonically in the vulva muscles, and these will often develop abnormally in the mutant. These two observations suggest that *ten-1 *may act cell-autonomously during the morphogenesis of hypodermal and vulva muscle cells. The *ten-1 *homologs in *Drosophila *and vertebrates are also expressed in embryos at sites and times of morphogenetic cell movements, and this is possibly an ancestral function for the gene [7,8,24,25].

### *ten-1 *participates in the development of all examined pharyngeal neurons

Only the *ten-1a *form is expressed in the pharynx, with expression in the marginal cells mc1 and mc3, and in the neurons M2L and M2R. However, all pharyngeal neurons examined exhibit defects in the *ten-1 *mutant. This suggests that the marginal cells mc1 and mc3 play an important role during pharyngeal neuron development. This is a reasonable hypothesis since the posterior part of mc1 occupies the metacorpus and mc3 occupies the posterior bulb, which are the two regions where most of the neuronal defects were observed. Another interesting possibility is that the two M2 neurons influence the development of the other pharyngeal neurons. The M2 neurons each send a long straight axon trajectory through the isthmus that develops without growth cones, and it is possible that it provides a pioneer axon function [26]. Other pharyngeal axons could grow through the isthmus by using growth cones that navigate along the M2 axon. In the absence of *ten-1*, these growth cones may stall, halt permanently or err in incorrect directions, which could explain the observed axon defects in and outside the isthmus for several of the pharyngeal axons studied (e.g. the M3, M4 and NSM neurons). Consistently, no varicosities or trajectory defects are ever observed within the isthmus for the M2 neurons.

### *ten-1 *interacts with several morphogenesis/axon guidance genes

*ten-1 *is synthetic lethal with mutations in the genes *sax-3*, *unc-34 *and *unc-73*. These genes are important for several axon guidance decisions during *C. elegans *development: *sax-3 *is the receptor for the guidance molecule *slt-1 *[27-29], and *unc-34 *and *unc-73 *regulate cytoskeleton dynamics in growth cones [30-33]. All three genes are also important for morphogenetic processes in early development, as evidenced by the low penetrance body shape abnormalities seen in these mutants. However, *ten-1 *must usually provide a function that allows most embryos to develop successfully in the absence of any one of these three genes. Chiquet-Ehrismann and co-workers have shown that *ten-1 *is important for organizing the ECM [13], which leads us to interpret our results in the following way. On the one hand, the single mutants of *sax-3*, *unc-34 *or *unc-73 *have defects in the regulation of cytoskeletal dynamics within cells undergoing morphogenesis but retain enough activity to succeed with this process provided that the extracellular environment is not also compromised. Conversely, the *ten-1 *mutant has defects in the composition/distribution of extracellular matrix important for morphogenetic processes but retains enough of it to complete embryogenesis provided that the ability of the cells to regulate their cytoskeletal dynamics is not compromised. Double mutants fall below essential thresholds and therefore fail. The most obvious developmental failure in *ten-1 *mutants is their deformed posterior half (Figs. 5 and 6), which correlates with the hypodermal cells failing to intercalate properly and to drive the convergent extension and contraction-driven elongation of which they are responsible [34,35]. The enhancement of the embryonic morphogenesis phenotype observed in the *ten-1 *and *sax-3, unc-34 *or *unc-73 *double mutants implicates all four genes in posterior hypodermal morphogenesis. Both *ten-1a *and *ten-1b *are expressed in the hypodermal cells of the posterior half, and a cell-autonomous function for *ten-1 *may explain why the posterior half is more susceptible to developmental failure.

It is worth noting that mutations in several genes have previously been reported to be synthetic lethal with the *ten-1(ok641)*: *dgn-1 *(dystroglycan), *nid-1 *(nidogen), *epi-1 *(laminin alpha-beta) and *ina-1 *(integrin) [13]. Pursuing with the above reasoning, it would appear again that two essential forces are in play: ECM integrity on the one hand (involving dystroglycan, nidogen and laminin) and cellular interaction/response to the ECM on the other (involving the integrin gene *ina-1*). When the ECM is too compromised, as in double mutants involving *ten-1 *and an ECM component gene, the embryo cannot undergo normal morphogenesis and is not viable. Conversely, when a partially compromised extracellular matrix mutant (*ten-1*) is combined with a mutation affecting interaction/response to the ECM (such as *ina-1*) then that too is not viable.

The observation that the *ten-1 *mutation also enhanced the penetrance of M2 neuron defects for the *unc-5*, *unc-129*, and *mig-14 *axon guidance pathways shows that *ten-1 *provides a function important for all these disparate pathways within the pharynx. Again, this function is likely to be related to providing a suitable ECM for the retention of guidance cues, and acting as a substrate for growth cone migration. The strong synergy observed between *ten-1 *and *unc-51 *is also interpreted in the same way: *unc-51 *is important for the transport of several guidance receptors on the plasma membrane of growth cones [21,36,37], and impairing both these receptors and the extracellular matrix that provides their cues and substrates has a severe impact on axon development.

### *ten-1 *genetically interacts with *unc-52 *and other extracellular matrix genes

Another dramatic enhancement in the frequency of M2 distal end defects was observed when the *ten-1 *mutation was combined with *unc-52*, which encodes the worm homolog of perlecan, an extracellular matrix component [22,38,39]. This result suggests that *ten-1 *and *unc-52 *act contribute redundantly to the formation of extracellular matrix suitable for the guidance of growth cones within the pharynx, just as *ten-1 *complements the activities of dystroglycan (*dgn-1*), laminin alpha beta (*epi-1*), nidogen (*nid-1*), collagen (*cle-1*) and perlecan *(unc-52*) in the extracellular matrix outside the pharynx [13].

### *ten-1 *is not generally essential for neuronal development

An apparent discrepancy between the present work and that of Chiquet-Ehrismann and co-workers regards extrapharyngeal neuron development. In contrast to their published observations [12], we were unable to document defects in neurons outside the pharynx, except for a low frequency (~2%) of L1 larvae having misplaced nerve ring axons and in a low frequency (<3%) of randomly picked L4 or adult worms that happened to have obvious hypodermal morphogenesis defects. It is important to note that the previously reported neuronal defects in *ten-1(ok641) *mutants were not quantified carefully, and in any case were most likely secondary to hypodermal morphogenesis defects.

### Neuronal phenotypes in mouse and worm teneurin mutants

Are the roles that *ten-1 *plays during pharyngeal neuron development evolutionarily conserved? That is obviously a difficult question. In the *C. elegans ten-1 *mutant, the defects in the pharyngeal neurons show much variation indicative of general pathfinding defects. This is consistent with the fact that *ten-1 *mutations typically enhance whatever defects are present in other mutants (e.g. *mig-14 or unc-5) *when mutations are combined. This appears to be in contrast with mouse teneurin mutants that exhibit quite specific path decision errors. Specifically, and as an example, the Ten-m3 mutant exhibits characteristic defects in ipsilateral, but not contralateral, guidance decisions during the development of the visual circuitry [40]. Indeed, Ten-m3 is expressed in a graded fashion consistent with a role as a guidance molecule for retinal fibers, and may guide their growth by mediating homotypic adhesion [41]. It therefore appears that at least some teneurin genes may guide neuronal development in mouse in ways that are more sophisticated than in nematodes.

## Conclusions

1. The novel allele *ten-1(et5) *is hypomorphic, which suggests that important post-embryonic function resides within the four EGF domains present in the *et5 *allele but absent from the null *ok641 *allele.

2. *C. elegans ten-1 *participates in the guidance of all tested pharyngeal neurons, and the *ten-1(ok641) *null mutation is synthetic lethal with mutations in cytoskeleton regulators (*sax-3, unc-34, unc-73*) and enhances the pharyngeal guidance defects of several mutations in axon guidance genes (e.g. *mig-14, unc-5, unc-51, unc-52 and unc-129*. *ten-1 *therefore complements these pathways during morphogenesis and axon guidance, perhaps by regulating the composition of the extracellular matrix.

3. *ten-1 *is not generally essential for neuronal development since neuronal defects outside of the pharynx were only rarely observed in *ten-1 *null mutants.

## Methods

### Strains

Worms were maintained at 20°C using standard methods [42]. The Bristol N2 strain was used as wild-type reference [43], and all strains were obtained from the *C. elegans *Genetics Center (St-Paul, Minnesota), unless stated otherwise. The following mutations were studied:

LG I: *unc-40(e271), unc-73(e396)*,

LG II: *mig-14(ga62), unc-52(e1421)*

LG III: *dpy-17(e164), mnm-5(et5), ten-1(ok641), ten-1(tm651), unc-32(e189)*

LG IV: *rac-2(ok326), unc-5(e53), unc-129(ev554)*

LG V: *unc-34(e315), unc-51(e369)*

LG X: *sax-3(ky123), slt-1(e15), unc-6(ev400)*

### Transgenes

In some cases, the plasmid *pRF4*, containing *rol-6(su1006) *which causes a Roller phenotype, was used as a transformation marker [44].

The following transgenes were used:

*etIs1 *and *etIs2 *which carry a *ric-19::gfp *translational reporter as well as pRF4, integrated into linkage groups IV and III, respectively [14].

*evIs79*, which carries *unc-129::gfp *[18]. This was a gift from Joe Culotti.

*jcIs1*, which consists of *pJS191 *(*ajm-1::gfp*), *pRF4 *and *C45D3 *(*unc29(+)) *DNAs [45].

*zdIs13*, which carries a *tph-1::gfp *transcriptional reporter expressed in the NSM pharyngeal neuron and HSN extrapharyngeal neuron [46]. This was a gift from S. Clark.

*muIs32*, which carries a *mec-7::gfp *transcriptional reporter expressed in the mechanosensory neurons. This was a gift from Cynthia Kenyon.

*etEx106*, which is an extrachromosomal array carrying a *ser-7b::gfp *transcriptional reporter [47] expressed in the M4 neurons as well as the plasmid pRF4.

### Sequencing of the *ten-1(et5) *mutation

Six fragments that covered the entire *ten-1 *gene were PCR amplified with *PFU Ultra *(Stratagene) on single lysed *mnm-5(et5) *or wild type worms as templates with the following primers: ten-1_fragment1_for: 5'-cgccgtcgtctgtgttcgaaac-3'+ ten-1_fragment1_rev: 5'-caaagctcctcaagaactactac-3', ten-1_fragment2_for: 5'-ctagtaacagatgatgaggcggc-3'+ ten-1_fragment2_rev: 5'-cgattcaccttcgaagtcttaggc-3', ten-1_fragment3_for: 5'-catggaagcaataagagccatatc-3'+ ten-1_fragment3_rev: cgcaccgttttagaattggtgac-3', ten-1_fragment4_for: 5'-ctaatgcgaaaggaggcagaagcc-3'+ ten-1_fragment4_rev: 5'-cagtctaccgaatcccaacctgac-3', ten-1_fragment5_for: 5'-ctgtaatggaaggggacgatgtgac-3'+ ten-1_fragment5_rev: 5'-caaactgccatccgaatcatcacc-3, ten-1_fragment6_for: 5'-cgtgatagggaatattggagactc-3'+ ten-1_fragment6_rev: 5'-cgttcacgccaccgacaaaatgtc-3'. The products were cloned into the *pCR-BluntII-TOPO *vector (Invitrogen) and sequenced by MWG Biotech (Germany).

### Generation of transgenic animals

Germline transformation was performed as described by Mello et al. 1991 and the dominant *rol-6 (su1006) *was used as a marker for transgenic worms, [44]. Plasmids were prepared with a Qiagen miniprep kit (Qiagen) and used with the following concentrations: *pRF4 (rol-6) *of 50 ng/μl, test plasmids of 25 ng/μl, and *pBSKS *(Stratagene) of 25 ng/μl.

### Detection of the *ten-1 *mutant alleles

When generating double mutants or lines carrying transgenes, it was often necessary to rely on PCR to detect the *ten-1 *alleles. This was done as follows. The *ten-1*(*ok641) *deletion was detected by PCR with TAQ DNA polymerase (Roche), using the following primers: forward-16: 5'-caccgttactaagccttcacgg-3'and reverse-8: 5'-ccactggaaaacgattgaggttt-3'which produces a product of 3 kb in wild type and 1 kb in the deletion mutant. The absence of wild type sequence in the mutant was confirmed with the following primers: forward-18: 5'-cttcgagtcattgccaattcaag-3'and reverse-8 which produces no band in the deletion mutant and a band of 1 kb in wild type.

Similarly, the *ten-1(tm651) *deletion was detected by PCR with TAQ DNA polymerase (Roche), using the following primers: forward-15: 5'-cagacctcatacgtctggaggagc-3' and reverse-10: 5'-cgtccgaacctgttggagatcc-3' which produces a product of 2350 bp in wild type and 1463 bp in the deletion mutant. The mutant with absent wild type sequence was confirmed with following primers: forward-16: 5'-caccgttactaagccttcacgg-3' and reverse-11: 5'-caactcggcttcgttcgttgat-3' which produces no band in the deletion mutant and a band of 280 bp in wild type.

### Construction of plasmids

Lysed wild type worms were used as template for PCR, amplifications were performed with *PFU Ultra *(Stratagene), the PCR products were gel purified with Qiagen Gel Extraction Kit (Qiagen), subcloned into the *pCR-BluntII-TOPO *vector (Invitrogen) and further transferred into *pPD95.77 *(Addgene).

#### pten-1a:gfp

This plasmid was created by amplification of 5520 bp 5'-UTR of the *ten-1 *long form, *ten-1a*, with the following primers: ten-1a_gfp_forward: 5'- **cgcatg**ccgttcattttccgtgtcaac-3' (SphI site in bold) and ten-1a_gfp_reverse: 5'-c**ctgcag**attaggcggtgggccttgc-3' (PstI site in bold). The PCR product was subcloned into the SphI and PstI sites of *pPD95.77*.

#### pten-1b:gfp

This plasmid was constructed by amplification of 3246 bp 5'-UTR of the *ten-1 *short form, *ten-1b*, with the following primers: ten-1b_gfp_forward: 5'-**cgcatg**cccatatgtctcttagtttagc-3'(SphI site in bold) and ten-1b_gfp_reverse: 5'-c**ctgcagg**gatcaccattgttcatagtgc-3' (PstI site in bold) and the PCR product was subcloned into the SphI and PstI sites of *pPD95.77*.

### Scoring of Neurons

#### M2 neuron

These neurons were visualized using *etIs2*, which carries a *ric-19::gfp *translational reporter expressed in the M2 neurons [14], or by using microinjection to generate transgenic lines carrying extrachromosomal arrays bearing both the pRIC-19::GFP [48] and pRF4 plasmids, and maintaining the transgenic lines by picking rollers.

#### M3 neuron

Transgenic lines carrying extrachromosomal arrays bearing the plasmid pQC105 and pRF4 were used to monitor the M3 neurons. pQC105 carries a *mnm-2::gfp *transcriptional reporter that is strongly expressed in the M3 neurons [49].

#### M4 neuron

Transgenic lines carrying extrachromosomal arrays bearing the plasmid *pser-7b::gfp *(a transcriptional reporter for *ser-7b; *[47]) and pRF4 were used to monitor the M4 neurons.

#### I3 neuron

Transgenic lines carrying extrachromosomal arrays bearing the plasmids *pten-1a:gfp *and pRF4 were used to monitor the I3 neurons.

#### Ventral motor neurons

The DA/DB motor neurons were visualized using *evIs79*, which carries *unc-129:gfp*. The expression levels of this reporter were also quantified with the free software ImageJ (Rasband, WS. ImageJ, U.S. National Institute of Health, Bethesda, Maryland, USA http://rsb.info.nih.gov/ij/).

#### Mechanosensory neurons

These neurons were visualized using the *muIs32 *transgene, which contains the *mec-7::gfp *transcriptional reporter.

#### Viability assay

For each genotype tested, adults were allowed to lay eggs during a period of 2 hours. Ten eggs were then gently transferred to each of ten plates. The worms from these plates were monitored daily for viability and any visible phenotype, and were transferred daily to fresh plates beginning from day three.

## Authors' contributions

CM and MP designed most of the experiments. CM mapped the et5 allele and identified the mutation. CM, VV and MP constructed most of the strains, characterized their phenotypes and scored the various pharyngeal and body neurons. GJ characterized the expression profiles of the GFP reporters. MP wrote the manuscript. All authors read and approved the final manuscript.

## Supplementary Material

Additional file 1 **Supplementary Figures**. **Fig. S1**. Extrapharyngeal neurons are normal in the *ten-1(ok641) *mutant. The pan-neuronal *evIs111 *transgene was used to visualize the entire nervous system in (A) wild-type or (B) *ten-1(ok641) *mutant adults. No trajectory defects or abnormal varicosities were observed in the mutant. Asterisks indicate cell bodies that are analogously positioned in wild-type and mutant: any differences are within the variation range found in wild-type. The *mec-7::gfp *transgene was also used to score the mechanosensory neurons in (C) wild-type and in (D) *ten-1(ok641) *mutant adults. Again, no differences were observed between wild-type and mutant animals (N>100 worms examined for each treatment). Meaning of lowercase annotations: vul (vulva), vnc (ventral nerve cord), tg (tail ganglion), nr nerve ring). **Fig. S2**. Body neurons expressing *ten-1b::gfp *only rarely exhibit defects in the *ten-1(ok641) *mutant. In young larvae (L2), there is evidence of incomplete fasciculation in the ventral cords of both wild-type and *ten-1(ok641) *worms (arrows in A-B). By the L4 stage wild-type and mutants have nicely fasciculated ventral nerve cords (arrows in C-D), and two well defined lateral axons on each side (arrowheads in C-D indicate one such pair). Only occasionally (<3%; N > 100) are abnormalities observed in the neurons of randomly picked L4s or adults, and this is fond only in those animals with deformed body shapes. The upper worm in (E) is a pregnant hermaphrodite with a deformed posterior half, and several errant axon trajectories, two of which are indicated by arrowheads. The worm just below it is also a mutant but has a perfectly normal nervous system. Scale bars represent 50 μm. Click here for file
