# Supplementary material for: C. elegans ten-1 is synthetic lethal with mutations in cytoskeleton regulators, and enhances many axon guidance defective mutants
Source: BMC Dev Biol. 2010 May 24;10:55. doi: 10.1186/1471-213X-10-55 (PMC2887410; doi:10.1186/1471-213X-10-55)

Fig. S1. Extrapharyngeal neurons are normal in the *ten-1(ok641)* mutant. The pan-neuronal *evIs111* transgene was used to visualize the entire nervous system in (A) wild-type or (B) *ten-1(ok641)* mutant adults. No trajectory defects or abnormal varicosities were observed in the mutant. Asterisks indicate cell bodies that are analogously positioned in wild-type and mutant: any differences are within the variation range found in wild-type. The *mec-7::gfp* transgene was also used to score the mechanosensory neurons in (C) wild-type and in (D) *ten-1(ok641)* mutant adults. Again, no differences were observed between wild-type and mutant animals (N>100 worms examined for each treatment). Meaning of lowercase annotations: vul (vulva), vnc (ventral nerve cord), tg (tail ganglion), nr nerve ring).

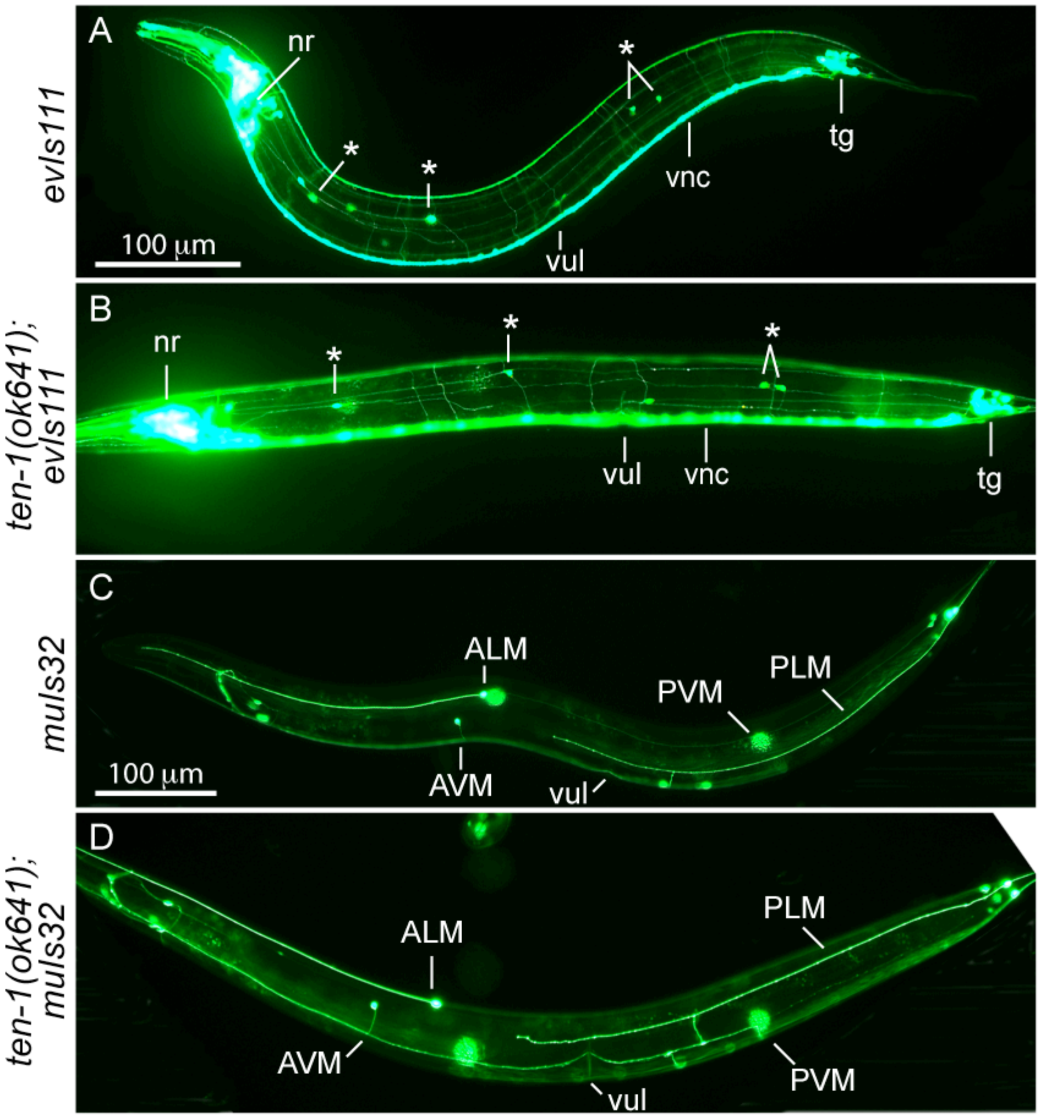

Fig. S2. Body neurons expressing *ten-1b::gfp* only rarely exhibit defects in the *ten-1(ok641)* mutant. In young larvae (L2), there is evidence of incomplete fasciculation in the ventral cords of both wild-type and *ten-1(ok641)* worms (arrows in A-B). By the L4 stage wild-type and mutants have nicely fasciculated ventral nerve cords (arrows in C-D), and two well defined lateral axons on each side (arrowheads in C-D indicate one such pair). Only occasionally (<3%; N>100) are abnormalities observed in the neurons of randomly picked L4s or adults, and this is fond only in those animals with deformed body shapes. The upper worm in (E) is a pregnant hermaphrodite with a deformed posterior half, and several errant axon trajectories, two of which are indicated by arrowheads. The worm just below it is also a mutant but has a perfectly normal nervous system. Scale bars represent 50 μm.

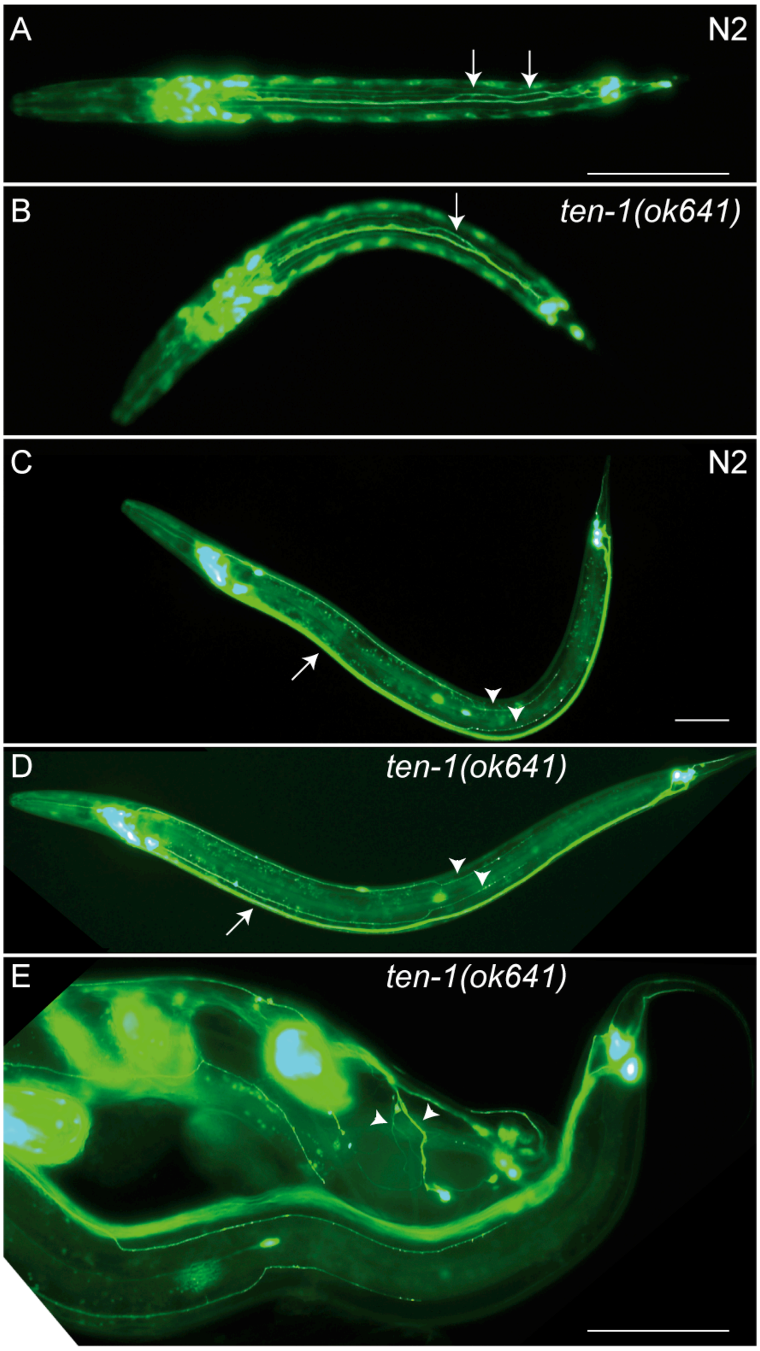

Supplement: Additional file 1 — Supplementary Figures. Fig. S1. Extrapharyngeal neurons are normal in the ten-1(ok641) mutant. The pan-neuronal evIs111 transgene was used to visualize the entire nervous system in (A) wild-type or (B) ten-1(ok641) mutant adults. No trajectory defects or abnormal varicosities were observed in the mutant. Asterisks indicate cell bodies that are analogously positioned in wild-type and mutant: any differences are within the variation range found in wild-type. The mec-7::gfp transgene was also used to score the mechanosensory neurons in (C) wild-type and in (D) ten-1(ok641) mutant adults. Again, no differences were observed between wild-type and mutant animals (N>100 worms examined for each treatment). Meaning of lowercase annotations: vul (vulva), vnc (ventral nerve cord), tg (tail ganglion), nr nerve ring). Fig. S2. Body neurons expressing ten-1b::gfp only rarely exhibit defects in the ten-1(ok641) mutant. In young larvae (L2), there is evidence of incomplete fasciculation in the ventral cords of both wild-type and ten-1(ok641) worms (arrows in A-B). By the L4 stage wild-type and mutants have nicely fasciculated ventral nerve cords (arrows in C-D), and two well defined lateral axons on each side (arrowheads in C-D indicate one such pair). Only occasionally (<3%; N > 100) are abnormalities observed in the neurons of randomly picked L4s or adults, and this is fond only in those animals with deformed body shapes. The upper worm in (E) is a pregnant hermaphrodite with a deformed posterior half, and several errant axon trajectories, two of which are indicated by arrowheads. The worm just below it is also a mutant but has a perfectly normal nervous system. Scale bars represent 50 μm. [file 1471-213X-10-55-S1.PDF]
